# PCOS Phenotype in Unselected Populations Study (P-PUP): Protocol for a Systematic Review and Defining PCOS Diagnostic Features with Pooled Individual Participant Data

**DOI:** 10.3390/diagnostics11111953

**Published:** 2021-10-21

**Authors:** Sylvia Kiconco, Aya Mousa, Ricardo Azziz, Joanne Enticott, Larisa V. Suturina, Xiaomiao Zhao, Alessandra Gambineri, Fahimeh Ramezani Tehrani, Bulent O. Yildiz, Jin-Ju Kim, Helena J. Teede, Anju E. Joham

**Affiliations:** 1 Monash Centre for Health Research and Implementation, School of Public Health and Preventive Medicine, Monash University, Clayton, VIC 3800, Australia; sylvia.kiconco@monash.edu (S.K.); joanne.enticott@monash.edu (J.E.); helena.teede@monash.edu (H.J.T.); 2 Departments of Obstetrics, Gynaecology and Medicine, School of Medicine, University of Alabama at Birmingham, Birmingham, AL 35294, USA; razziz@uabmc.edu; 3 Department of Healthcare Organization and Policy, School of Public Health, University of Alabama at Birmingham, Birmingham, AL 35294, USA; 4 Department of Health Policy, Management and Behaviour, School of Public Health, University at Albany, SUNY, Rensselaer, Albany, NY 12144, USA; 5 Department of Reproductive Health Protection, Scientific Center for Family Health and Human Reproduction, 664003 Irkutsk, Russia; lsuturina@mail.ru; 6 Reproductive Endocrinology and Infertility Sun Yat-sen Memorial Hospital, Sun Yat-sen University, Yanjiang Road 107, Guangzhou 510120, China; zhaoxmiao@163.com; 7 Department of Medical and Surgical Science, University of Bologna, 40138 Bologna, Italy; alessandra.gambiner3@unibo.it; 8 Reproductive Endocrinology Research Center, Research Institute for Endocrine Sciences, Shahid Beheshti University of Medical Sciences, Tehran P.O. Box 19395-4763, Iran; ramezani@endocrine.ac.ir; 9 Division of Endocrinology and Metabolism, Hacettepe University of Medicine, Ankara 06100, Turkey; byildiz@hacettepe.edu.tr; 10 Division of Reproductive Endocrinology, Department of Obstetrics and Gynecology, Healthcare System Gangnam Center, Seoul National University Hospital, Seoul 03080, Korea; kimjinju0514@gmail.com; 11 Department of Endocrinology and Diabetes, Monash Health, Clayton, VIC 3168, Australia

**Keywords:** PCOS, polycystic ovaries, Stein-Leventhal, epidemiology, normative cut-offs, phenotype, cluster analysis, hirsutism, androgens, menstrual cycle, PCOM

## Abstract

The diagnosis of polycystic ovary syndrome (PCOS) remains challenging due to limited data regarding normative cut-offs for the diagnostic features in different subpopulations. We aim to conduct a systematic review, build a comprehensive repository of de-identified individual participant data (IPD), and define normative ranges and diagnostic cut-offs for all PCOS diagnostic features. We will conduct a systematic search of MEDLINE and EMBASE databases for studies that assessed PCOS diagnostic features in unselected women. Two reviewers will assess eligibility and perform quality appraisal. Authors of included studies will be invited to contribute IPD. Primary variables include directly assessed modified Ferriman Gallwey (mFG) scores; menstrual cycle lengths; follicle number per ovary (FNPO), ovarian volume (OV), anti-Müllerian hormone (AMH); circulating androgens, including total testosterone (TT), free testosterone, bioavailable testosterone, free androgen index (FAI), androstenedione (A4), and dehydroepiandrosterone sulphate (DHEAS). Normative ranges and cut-offs will be defined using cluster analysis. Monash University Human Research Ethics Committee granted ethical approval (26938/0 1/12/2020), all IPD will be de-identified and primary studies have ethical approval from their institutional ethics committees. Findings will clarify distinction between PCOS and non-PCOS populations, and inform the update of the international evidence-based guidelines for the assessment and management of PCOS.

## 1. Introduction

Polycystic ovary syndrome (PCOS) is a complex endocrine disorder affecting 8% to 13% of reproductive-aged women globally [1], making it a key public health burden. Women with PCOS are at an increased risk of adverse reproductive, metabolic, psychological, oncological, pregnancy, and long-term offspring metabolic and developmental disorders, which impair their quality of life [2,3] in addition to straining health and economic resources [4]. Despite the adverse outcomes associated with PCOS, it is estimated that up to 70% of PCOS cases remain undiagnosed [5]. The diagnosis of PCOS can be complicated by several factors including the current challenges in defining individual clinical features within the diagnostic criteria; clinical heterogeneity resulting in various phenotypes; ethnic differences, and variations in clinical features across the life course. These challenges often result in delayed diagnosis and dissatisfaction with care, as widely reported [6,7].

The current international evidence-based guidelines for PCOS diagnosis and management [8,9] recommend the use of the 2003 Rotterdam criteria [10], which require the presence of any two of these features: (a) oligo-ovulation and/or anovulation, (b) biochemical or clinical hyperandrogenism, and (c) PCOM. Thresholds for each of these diagnostic features have been defined, but the cut-offs are often based on an arbitrary percentile (e.g., 95th percentiles) from variably defined populations and dependent on specific laboratory ranges as defined by the assay manufacturer [8,11,12]. Additionally, diagnostic features represent a continuum across the lifespan [13,14]. This makes it difficult to distinguish between women with PCOS and those without, due to overlapping features.

To determine normal values for PCOS features, it is recommended that investigators prospectively establish how parameter values are distributed naturally within their populations [15]. For the purpose of assessing a large unselected well-characterized population, cluster analysis is the preferred method as it considers the presence of distinct natural groups or ‘clusters’ to determine normative ranges. The use of an uppermost normal centile as the maximum level of a parameter for a specific population in a ‘super control’ cohort is also a recommended approach, although the biological basis for this approach is unclear.

Notwithstanding, the majority of studies in PCOS use summary measures such as means and standard deviations or arbitrary percentiles to define cut-off values for abnormal or normal ranges. These measures limit understanding of the individual inherent features of PCOS. A small number of studies have attempted to define normal ranges for hirsutism [16,17], PCOM [18] and androgens [19,20] using cluster analysis, but features vary between study settings and background population characteristics. It is thus crucial to determine population-specific normative ranges for each of the PCOS diagnostic features in order to standardize the differences between women with and without PCOS across regionally distinct populations.

This protocol describes the methodological approach by which we will collect and analyze international multi-ethnic data from well-characterized unselected (medically unbiased) populations of women to define normative cut-offs for menstrual cyclicity, clinical and biochemical hyperandrogenism, and ovarian morphology. The data repository of individual participant data (IPD) generated will be an invaluable resource in answering important research questions, which will assist in improving our understanding of PCOS aetiology and phenotype.

The primary research questions underpinning the study include; (1) What are the normal ranges and cut-off values for menstrual cyclicity in women with and without PCOS? (2) What are the normal ranges and cut-off values for defining hirsutism (modified Ferriman-Gallwey [mF-G] scores) in women with and without PCOS? (3) What are the normal ranges and cut-off values for circulating androgens, including total testosterone (TT), free testosterone, bioavailable testosterone, free androgen index (FAI), androstenedione (A4), and dehydroepiandrosterone sulphate (DHEAS), in women with and without PCOS? (4) What are the normal ranges and cut-off values for ovarian markers, including follicle number per ovary (FNPO)/antral follicle count (AFC), ovarian volume (OV), and anti-Müllerian hormone (AMH) levels in women with and without PCOS? Secondary research questions will explore the minimum number of assessment areas for hirsutism, associations between diagnostic criteria and other outcomes of PCOS as outlined in the PCOS core outcomes set [3], as well as differences across androgen assessment methods, ethnic groups, and age ranges. Establishing association between PCOS diagnostic criteria and the outcomes as per the PCOS outcomes set is crucial in identification of individuals at risk and the need for monitoring.

## 2. Experimental Design and Procedure

### 2.1. Selection of Studies and Participants

Systematic review to identify research groups and data for inclusion: The initial systematic review will be undertaken to identify research groups and data for inclusion, and will be conducted according to the Preferred Reporting Items for Systematic Reviews and Meta-analyses of IPD Statement (PRISMA-IPD) [21]. The protocol for this review and IPD analysis has been registered with PROSPERO (CRD 42021267847).

Literature search strategy: To identify eligible studies, we will conduct a systematic search of EMBASE and MEDLINE databases using a specified search strategy. A manual Google search and scanning of bibliographies and citations of relevant studies will be performed to identify additional studies. Collaborators will also be consulted to identify any potentially eligible ongoing or unpublished studies.

Selection criteria: Based on the selection criteria outlined in Table 1, the titles and abstracts of studies identified from the search will be assessed by one reviewer (SK) in consultation with two senior researchers (RA and HT). Seemingly eligible studies will then be screened in full text by two independent reviewers (SK and AM) who will also perform quality appraisal. Study screening and selection will be conducted using Covidence software. The suggested minimum study sample size of ≥300 for each study is based on an estimated PCOS prevalence of at least 10%, with a precision of at least 2% at 95% confidence interval [22]. Corresponding and/or lead authors of identified studies will be contacted and asked to participate and contribute de-identified IPD for integration and analysis. It is expected that the population in each study will be well-profiled in terms of various attributes including ethnicity, age, body mass index (BMI), and the use of contraceptive pills or other hormonal treatments.

#### Specific Definitions for PCOS Diagnostic Features

This project will include data from studies identified in the systematic review and whose researchers agree to participate, where PCOS was directly assessed and reported using the 1990 NIH, the 2003 Rotterdam criteria, and the 2006 Androgen Excess & PCOS Society [AE-PCOS] criteria [10]. To be included and integrated, data regarding PCOS features must have been assessed as described below.

PCOM: PCOM is assessed by the presence of enlarged ovaries with numerous peripheral developing follicles and increased stroma (stromal hypertrophy) on ultrasound [23]. As such, follicle number per ovary (FNPO) and ovarian volume (OV) are the preferred parameters to assess PCOM, as they can be measured in real time and are consistent features of polycystic ovaries [24]. The international PCOS guideline does not recommend PCOM assessment for PCOS diagnosis in women of less than eight years post-menarche [8]. This study will integrate PCOM data that were generated using recommended assessment routes, i.e., transvaginal or endovaginal ultrasound for FNPO and transvaginal, endovaginal, or transabdominal ultrasound for OV, including specifications regarding the bandwidth frequency of the transducer used. Data related to PCOM assessment reporting should essentially include total FNPO measuring 2–9 mm, three dimensions and volume of each ovary, endometrial thickness and appearance, and any ovarian and uterine pathology, including ovarian cysts, corpus luteum, and/or dominant follicles ≥ 10 mm [8]. These assessments should have been conducted and reported by specially trained personnel.

In addition to FNPO and OV, mounting evidence suggests that AMH may be a putative biomarker for PCOM [18,25]. AMH is a glycoprotein secreted by the granulosa cells of small, growing (antral) follicles [26] and elevated levels in women with PCOS are reportedly associated with high follicle number and hypersecretion of the granulosa cells [27,28]. Therefore, available AMH data that was generated using validated assays will also be integrated in the IPD analysis.

Clinical and biochemical hyperandrogenism: Hyperandrogenism can be assessed either clinically or biochemically. Clinical hyperandrogenism includes hirsutism, alopecia, and acne. Hirsutism and alopecia are assessed using the modified mF-G and Ludwig visual scores, respectively, but there is no internationally accepted visual tool for the assessment of acne [9]. This study will integrate hirsutism data as assessed using the mF-G score.

To assess biochemical hyperandrogenism (i.e., hyperandrogenemia) recommended androgen indices for assessment in PCOS include directly assessed total testosterone, free testosterone (FT), calculated free testosterone (cFT), or calculated bioavailable testosterone (cBT) and calculated FAI. Use of high-quality laboratory assays such as chromatography immunoassays and liquid chromatography-mass spectrometry (LC-MS) is recommended instead of using direct free testosterone assays, such as radiometric or enzyme-linked assays [8,9]. Data on androstenedione and DHEAS levels will also be considered for phenotypic completeness. In addition to the quality of assays, the timing of androgen status assessment will also be considered, as testosterone secretion may be increased during mid-cycle. Data from participants who were using hormonal contraception and/or had not withdrawn from contraception or medication use for at least three months before assessment will be excluded from the analysis related to biochemical hyperandrogenism.

Ovulatory dysfunction: In PCOS diagnosis, ovulatory dysfunction is usually reflected by clinically overt menstrual cycle irregularity, which is defined as reported vaginal bleeding occurring > 35 days or <21 days intervals [8]. In addition to assessing menstrual cycle lengths, participants may be assessed by evaluating the number of times they have had their menstrual periods over a 12-month period. Some patients with ovulatory dysfunction will exhibit what appears to be eumenorrhea (i.e., regular episodes of vaginal bleeding) and in these women ovulatory dysfunction will be detected by the measurement of a progesterone level in the luteal phase of the cycle.

### 2.2. Data Sharing and Safety

A single secured network repository (database) will be built by integrating IPD from all included studies following a standardized format. A data access and sharing policy will be developed with input from all study collaborators or authors of the included studies who contribute IPD. To ensure safety of data, the Monash-Helix based encrypted secure file transfer protocol (SFTP) platform will be used to transfer data to the repository. Data and relevant dictionaries will be shared and accepted in any available electronic formats. The SFTP platform is user-friendly and employs a multitude of controls to protect data from unauthorized access. These controls are regularly audited to ensure they meet global best practices and are aligned with ISO 27001 security practices [29].

Upon being uploaded into the repository, all data will be cross-checked to ensure correct and harmonized coding, identify missing data, and any issues will be queried as necessary with the data custodians. Data for each PCOS phenotypic feature from the different studies will be converted into consistent units where necessary. Further IPD analysis between participating collaborators and other researchers will be authorized by the steering committee and the Secure eResearch Platform (SeRP) will be used to access and analyze the relevant data. SeRP is also ISO27001-certified [30] and allows only the authorized data owner or data custodian to view and control how data is used by other authorized researchers. A data access and sharing policy will be developed with input from all study collaborators and authors of the included studies who contribute IPD. This will allow data access to all team members and other eligible researchers.

Data variables and extraction: Key variables and corresponding IPD to be extracted from each study include study and participant characteristics, as well as values for each PCOS diagnostic feature as described above [8,9,31]. A list of minimum variables to be extracted from each study is shown in Table 2. Further, because the research focus of these studies may have evolved over time, other PCOS endpoints as described in the PCOS core outcomes set [3] will be extracted if they were collected in the primary study. Specific endpoints will include; type 2 diabetes, insulin resistance, impaired glucose tolerance, hypertension, coronary heart disease, lipid profile, venous thromboembolic disease, pregnancy viability, live birth, miscarriage, stillbirth, neonatal mortality, gestational weight gain, gestational diabetes, preterm birth, hypertensive disease in pregnancy, baby birth weight, major congenital abnormalities, depression, anxiety, eating disorders, abnormal endometrial proliferation, and endometrial cancer. Where data were collected, the association between quality of life and diagnostic criteria will also be assessed. The IPD will be integrated from all included studies to build a PCOS diagnosis data repository in a standardized and consistent manner to define PCOS phenotypes. All datasets will be reviewed to identify potential inconsistencies and any concerns will be resolved in consultation with the authors.

Risk of bias assessment: Risk of bias for the included studies providing IPD will be assessed independently by two reviewers. The appraisal tool (AXIS) for cross-sectional studies [32] will be used to rate each study as having a low, moderate, or high risk of bias. Appraisal domains will include selection criteria of participants, sampling methods, sample size and study power, methods of exposure and outcome assessment, data analysis methods, conflicts of interest among the authors, and reporting of results. Any appraisal disagreements will be resolved through discussion and consensus.

### 2.3. Data Analysis

Quality assessment: All data for the key PCOS diagnostic features are continuous, and means or medians with standard deviations, or 95% confidence intervals, will be extracted. Using Review Manager (RevMan v.5.3), heterogeneity will be assessed using the *I*^2^ statistic and publication bias will be tested visually via funnel plots and Egger’s test [33].

Cluster analysis: To define PCOS phenotype, all IPD analyses will be conducted in consultation with a senior biostatistician. Cluster analysis will be conducted using the *k*-means method [34] including other covariates such as BMI for each potential cluster to explore normative ranges and cut-offs for each PCOS diagnostic feature. Each of these features will be categorized from the 5th to 95th percentile and homogeneity of clusters will be assessed by the squared correlation ratio (R^2^). The R^2^ for each variable will also be computed to examine the most significant variables in identifying clusters. For each PCOS feature, the number of clusters will be informed using a graphical representation of R^2^ values against the overall number of clusters. The quality of cluster separation (cluster membership discrimination) will be determined using a k factorial discriminant analysis and receiver operating characteristics curves (ROC) will be constructed to examine the cut-off values for each feature between PCOS and non-PCOS groups [35]. Repeated, k-fold cross-validation will be used for validating each classifier. The cluster composition producing the highest area under the receiver operating characteristic curve (AUROC) in a given cohort will be chosen as the optimal clustering method. Other clustering methods will also be examined to explore the robustness of the findings. While k-means algorithms can be considered traditional clustering techniques, we will also explore the findings from new novel clustering algorithms such as PAM as a k-medoids algorithm—a viable alternative to k-means clustering and CLARA as an advancement of k-medoids algorithms compatible with relatively large datasets [36].

To assess the association between diagnostic criteria and secondary endpoints, logistic or linear regression models will be used for categorical and continuous variables respectively at 95% level of significance.

Subgroup and sensitivity analyses: Subgroup analyses will be performed for adult women and adolescent populations in both aggregate and IPD formats as PCOS features vary between different age groups. More subgroup or meta-regression analyses for ethnicity, BMI, period since menarcheal age, and PCOS criteria will also be conducted. Further, in circumstances where different assay types have been used for the same variable such as testosterone, subgroup analyses will be considered. Sensitivity analyses will be performed where the *I*^2^ is > 70% as well as by risk of bias to examine whether studies with a high risk of bias are influencing the overall results [37]. Studies not contributing IPD will also be explored in these sub-analyses to detect any data availability bias.

## 3. Expected Results

Following the PRISMA reporting guidelines, results will be presented in summary tables and corresponding narrative formats including funnel plots from the publication bias assessments.

Results from the IPD cluster analysis will be summarized in graphs and receiver operating characteristics curves for each PCOS diagnostic feature. Discriminant score plots will also be presented to show the quality of cluster separation.

Findings from this study will be used to inform the update of the international evidence-based guidelines for the assessment and management of PCOS for optimal diagnosis. This will be the first study to define PCOS diagnostic features and cut-offs using integrated data from multi-ethnic, unselected, and well-defined populations globally. Our findings will offer an opportunity to overcome the ethnic and age variation challenges surrounding the definition of PCOS diagnostic features. In addition, the use of cluster analysis provides the most robust approach in defining normal ranges and distinguishing populations [15], and an extensive search strategy to identify potential research groups to participate will be used.

Although various research groups may have used different procedures in the evaluation of these features previously, this study will use only data that were generated following the current guideline recommendations for each diagnostic feature [9]. Review and synthesis of raw data, standardization, and cleaning will be key at the initial stage of this study for consistency. Despite the rigorous methods planned, the potential for publication bias or data availability bias cannot be ruled out and may influence our results. However, collaborative research and the chosen study approach will likely minimize these methodological issues. Utilizing existing IPD offers a more efficient process for acquiring well-profiled, representative data in defining PCOS diagnostic features compared with conducting a new population-based study.

## 4. Ethics and Dissemination

The proposed study will be conducted in accordance with the guidelines of the Declaration of Helsinki and Ethical approval to collate and develop data from eligible studies was obtained from Monash University Human Research Ethics Committee (HUMREC) (ID: 26938). In addition, individual studies will have ethical approval from their respective Human Research Ethics Committees in the countries where the studies are being or have been conducted. All data will be completely anonymized prior to being imported into the repository and a new random identification number will be allocated to each participant to ensure complete de-identification. A data sharing agreement will be signed between the lead institution and each participating center through the main investigator(s) and/or nominated data custodian. The study will have a steering committee which comprises all members contributing IPD. The roles and responsibilities of the committee will be outlined in the terms of reference during invitation of eligible study authors to participate.

Findings from all analyses will be disseminated through various platforms including scientific meetings, workshops, conferences, and journals as deemed appropriate. Due to the collaborative nature of this project, authorship guidelines to streamline dissemination will be developed in consultation with all IPD contributors.

## Figures and Tables

**Table 1:** PICO table for the study selection criteria.

	Participants (P)	Intervention (I)	Comparison (C)	Outcomes (O)	Setting
Inclusion	Women of any age, ethnicity, or weight	Direct assessment of any of the PCOS features and/or end points	Women without any PCOS features	Primary outcomes -Ovulatory dysfunction: (Menstrual cycle lengths) -Clinical hyperandrogenism (Hirsutism (mFG scores)) -Biochemical hyperandrogenism: (Total testosterone, free testosterone, FAI) -PCOM: Ovarian volume, Follicle number per ovary Secondary outcomes -A_4_, DHEAS, AMH, SHBG	Unselected population such as school and employee databases
Exclusion	Pregnant populations or if women were on any hormonal treatment during assessment or (within 3 months pre-assessment), or cardiometabolic related comorbidity	Studies where these features are self-reported or used ICD Codes, or unverified registry data	n/a	Studies not reporting PCOS features and/or related endpoints	Selected or referred population such as a hospital
Study type		Cross sectional/prevalence, or longitudinal studies with sample size ≥ 300			
Language		No limit			
Year of publication		1990 onwards			

ICD, International classification of diseases; PCOS, polycystic ovary syndrome; PCOM, polycystic ovarian morphology; mFG, modified Ferriman Gallwey; SHBG, Sex hormone-binding globulin; FAI, Free androgen Index, A4, Androstenedione; DHEAS, Dehydroepiandrosterone sulphate; n/a, not applicable.

**Table 2:** IPD to be obtained from included studies.

Study Characteristics	Participants	Primary PCOS Features
First author, year, and country of publication	Age at assessment, ethnicity, education level, marital status	Menstrual cycle length (days) or number of menstrual periods per year
Study design, sample size	Smoking status, physical activity, alcohol consumption	Modified Ferriman Gallwey (mFG) score,
Selection criteria	Weight, height, or Body mass index (BMI), (kg/m^2^), waist circumference (cm), waist hip ratio,	Total testosterone (TT) *, Sex hormone-binding globulin (SHBG) *, Free androgen Index (FAI) *, Androstenedione (A4) *, Dehydroepiandrosterone sulphate (DHEAS) * with units and assay types used for each
Study duration and timing of data collection	Hormonal treatment (OCPs/HRT)	Ovarian volume (OV), cm^3^, Follicle number per ovary (FNPO)-including transducer frequency used, Anti-müllerian hormone (AMH) * with units and assay or test types used
-	-	Other features and outcomes as per the PCOS core outcomes set

OCPs, Oral contraceptive pills; HRT, hormone replacement therapy. * Units will be standardized/converted for consistency.

## Data Availability

No data have been generated in this protocol.
